# Work-Family Segmentation Preferences and Work-Family Conflict: Mediating Effect of Work-Related ICT Use at Home and the Multilevel Moderating Effect of Group Segmentation Norms

**DOI:** 10.3389/fpsyg.2019.00834

**Published:** 2019-04-16

**Authors:** Jing Yang, Yucheng Zhang, Chuangang Shen, Siqi Liu, Shanshan Zhang

**Affiliations:** ^1^College of Tourism, Huaqiao University, Quanzhou, China; ^2^School of Public Administration, Southwestern University of Finance and Economics, Chengdu, China; ^3^School of Business, Huaqiao University, Quanzhou, China; ^4^School of Nursing, Chengdu University of Traditional Chinese Medicine, Chengdu, China

**Keywords:** work-family segmentation preferences, segmentation norms, work-related ICT use at home, work-family conflict, multilevel analysis

## Abstract

Drawing on boundary theory, we propose a multilevel model that examines the effects of group segmentation norms on individual-level processes, relating segmentation preferences to work-family conflict via the use of a technological boundary. Data from 350 married employees in 81 working groups were used to test the model. The results of multilevel analysis revealed that work-related information and communication technology (ICT) use at home mediated the relationship between employee work-family segmentation preferences and work-family conflict, and the segmentation norms of the team moderated the relationship between work-family segmentation preferences and work-related ICT use at home. Managerial and practical implications are discussed.

## Introduction

Due to advances in modern information and communication technology (ICT), such as smart phones, e-mail, and social applications, dramatic changes have been observed in the workplace and daily life. ICT use at home makes employees believe that they must be available and accessible at all times (Major and Germano, 2006; Boswell and Olson-Buchanan, 2007; Middleton, 2007; Hendon et al., 2017), which increases their working time (Milliken and Dunnjensen, 2005; Higgins et al., 2006). Specifically, ICT use at home enables employees to connect to work during non-working hours, such as by allowing them to check and reply to e-mails related to work at night or on weekends. Considering that the use of ICT is such an important aspect of work in the electronic era, Gephart (2002) noted that scholars studying organizational behavior have redefined “work” with regard to ICT use in the modern electric age. Since ICT use involves both work and family domains, for more than a decade, researchers have examined the influence of ICT on work and life in order to clarify whether the role of ICT in work-family interfaces is positive or negative (Towers et al., 2006; Boswell and Olson-Buchanan, 2007; Kreiner et al., 2009).

However, there are two different views regarding ICT use at home in the work-family interface. Some researchers have suggested that ICT use benefits the interface of work and family roles, leads to an integrative boundary between work, and family. Specifically, it helps individuals quickly switch roles to meet the needs of different domains, which give them a higher sense of control over their work or family roles (Ninaus et al., 2015), and also helps them better fulfill their responsibilities and expectations of family roles (Derks et al., 2016). Besides, the integration of work-family boundary is conducive to the spillover of work satisfaction experienced by employees into their family domain, such as enhancing their marital satisfaction and emotional state at home (Ilies et al., 2009). However, most studies have found that although ICT use at home can improve sense of control over work or family roles, it also makes boundaries between work and family become more and more permeable, which leads to the boundaries becoming increasingly blurred (Kreiner, 2006). Blurred boundaries hinder individuals’ perception of domain roles and make them experience higher work-family conflict (Park et al., 2011; Gadeyne et al., 2018). Moreover, they also have impact on individuals’ work domain to some extent, resulting in high job burnout, and even turnover intentions among employees (Ferguson et al., 2016; Carlson et al., 2017).

In view of the influences of ICT use on work-family boundaries, it is possible an effective means of boundary management for individuals to choose whether to use ICT to meet the role needs of the other domain (Kossek et al., 2006; Olson-Buchanan and Boswell, 2006; Bulger et al., 2007), which may provide an alternative perspective to explain the inconsistent results mentioned above. According to boundary theory (Ashforth et al., 2000), work, and family are considered two relatively independent domains. When the boundaries between work and family domains are clear, the roles in both domains are segmented. On the contrary, when the boundaries are ambiguous, the roles are integrated, which results in role confusion, and work-family conflict. For example, some employees who prefer to use ICT at home to solve work problems may experience high work-family conflict. On the contrary, those who prefer not to use ICT at home but are required to remain available even during off-duty time may feel their family life is invaded, which results in low work-family conflict. Olson-Buchanan and Boswell (2006) found that individuals could create work-family boundaries by actively restricting their work-related ICT use at home, which is an effective strategy for boundary management. Employees’ adoption of work-family boundary management strategies is mainly dependent on individuals’ and their working groups’ work-home segmentation preference (Kreiner, 2006). Park and Jex (2011) have found that the relationship between segmentation preference and work-family interference was mediated by boundary creation around ICT use.

Considering individual and group preference, the purpose of current study is to explore whether individual segmentation preference and working group segmentation jointly affects ICT use simultaneously. Prior research have focused on the effect of organizational policies on the use of ICT. Recently, researchers began to pay more attention to the effect of working environment (Gadeyne et al., 2018). According to Kossek and Lautsch (2012), effective work-family boundary management should occur “*in situ*,” and is affected by individual and organizational factors and also their interaction. In other words, employees with same work-family segmentation preference showing similar behavior will have different experiences in different situations. Because situations can affect the interpretation of their behavior, the meaning they give and the experience they have (Zimmerman et al., 2016). When the group holds integration norm for work and family, members in the group are expected to bring work home, and keep available whenever necessary even at home (Kreiner, 2006; Derks et al., 2016). In this situation, for those with high segmentation preference, they may feel that they have lost their autonomy in using ICT during off-hours, thus may lead to a higher level of work-family conflict (Kossek and Lautsch, 2012). In contrast, when the group holds segmentation norm for work and family, members in group are expected to finish the work before leaving work, and are expected that work and family roles do not interfere with each other. Under these circumstances, employees with either segmentation or integration preference are more autonomous to ICT use (Kossek et al., 2006). Gadeyne et al. (2018) found that the relationship of work-related PC/laptop use and work-to-home conflict was moderated by individual integration preference and working environment (organizational integration norms and work demands). However, one limitation of this study was that it neglected individual integration preference and working environment were at different levels, which resulted in ecological fallacy.

In order to fully reveal the occurrence and influence mechanism of work-related ICT use at home, both individual- (e.g., segmentation preference) and group- (e.g., segmentation norm) level variables should be taken into account simultaneously. Therefore, the present study builds a multilevel model to examine the cross-level interactive effects between group segmentation norms and individual segmentation preferences on WFC by employees’ work-related ICT use at home.

This study attempts to make three contributions. First, our research detects a cross-level moderation effect of group segmentation norms in connection with the relationship among segmentation preference, work-related ICT use and work-family conflict. It attempts to explain the inconsistent findings on the influence of ICT use on work-family interference from a cross-level perspective, which extend prior related research that have not distinguished between individual- and group-level variables (Gadeyne et al., 2018). Second, this study highlights the mediating mechanism of ICT use based on boundary theory concerning how boundary creation regulates work-family conflict from a work-family segregation perspective, which deepens our understanding of boundary theory. Third, this study provides practical insights for both researchers and practitioners who hope to reduce employees’ stress associated with work-family interference.

### Work-Family Segmentation Preferences and Work-Related Use of ICT at Home

According to the boundary theory proposed by Ashforth et al. (2000), the boundaries between work and family affect the transfer of resources. Individuals can build different role boundaries based on their work and family lives to manage the activities related to each area. Role boundaries are expected to simplify and regularize their environment. The ease of the transition between the work and family roles, however, is dependent on the level of segmentation and integration of the two roles. Work-family segmentation preferences refer to the degree to which one prefers to separate various aspects of work and family from each other by creating more or less impermeable boundaries around the work and family domains (Kreiner, 2006).

Different individuals have different preferences regarding whether to segment or integrate their work and family roles. Employees who prefer to create and maintain a clear partition to separate their work and family domains could avoid the impact of their work issues on their families (Liu et al., 2013; Hislop et al., 2015). Overall, “segmenters” prefer to create highly impermeable boundaries to maintain separation between work and family, while “integrators” prefer to maintain highly permeable homework boundaries to blend work and family aspects (Kreiner, 2006; Powell and Greenhaus, 2010). The work-family segmentation preference directly affects how individuals manage work-family activities (Kreiner, 2006; Kreiner et al., 2009). As a result, individuals who strongly prefer work-family segmentation will create impermeable boundaries between their work and family so that they are not likely to be impacted by their work-related issues at home (Liao et al., 2016).

Powell and Greenhaus (2010) found that the work-family segmentation preferences of employees is positively related to actual segmentation of the work domain from the family domain, and actual work-family segmentation is negatively related to work-to-family conflict. However, they fail to explain how the work-family segmentation preferences lead to the WFC. Integrators with low work and family boundaries tend to use ICT for work during non-work hours, which might be more effective than segmentation in protecting their valuable personal time (Grant and Kinman, 2014). In contrast, because segmenters with high work and family boundaries prefer to strictly separate work and non-work time, they are more likely to see the use of work-related ICT as a loss of personal time (Allen et al., 2014; Gadeyne et al., 2018). Accordingly, individuals with strong segmentation preferences seek to retain independent work and family roles and are more likely to restrict the working use of ICT during their private time. Conversely, those with weak segmentation preferences are less likely to restrict the use of ICT at home when encountering the same types of tasks. Hence, we propose the following hypothesis:

**Hypothesis 1:** The work-family segmentation preferences of employees are negatively correlated with their work-related ICT use at home.

### Work-Related Use of ICT at Home and Work to Family Conflict

Work to family conflict (WFC) refers to the role-transition conflict that an individual experiences when the demands of work spill over into family (Greenhaus and Beutell, 1985). The use of ICT enables employees to fulfill their job-related tasks at all times, which binds them to their jobs, and prevents them from escaping the role of an employee, even at home (Fenner and Renn, 2004; Robinson, 2006). According to the conservation of resources (COR) theory developed by Hobfoll (1989), there are four resource categories, including object resources, condition resources, personal resources, and energy resources. The value of resources varies among individuals and is related to their personal experiences and situations. Resources have two spiral effects, including loss spiral, and gain spiral. The loss spiral indicates that individuals lacking resources are more vulnerable to the pressure, which further accelerates the loss of resources. The gain spiral indicates that the individuals with sufficient resources are more capable of obtaining resources, which further produces greater resource. However, as the formation of the gain spiral is often not as fast as the loss spiral, people who lack resources are more likely to fall into the loss of the spiral. Because time and energy are limited resources for individuals, when employees use work-related ICT at home, employees’ family time is reduced (Hobfoll and Shirom, 2001; Halbesleben et al., 2009, 2014). Thus, when employees realize that their resources may decrease and the required resources are beyond reach, they may feel stressed, and exhausted. When work-related ICT use encroaches on employees’ private time, they will experience high WFC. Based on these arguments, the following hypothesis is proposed:

**Hypothesis 2:** The frequency of the work-related use of ICT at home is positively correlated with the level of WFC.

### Work-Related Use of ICT at Home as a Mediator

According to boundary theory, an employee’s preference regarding work-family segmentation will affect his/her work-family interface (Ashforth et al., 2000; Matthews and Barnes-Farrell, 2010). Studies have suggested that employees with high work-family segmentation preferences will consciously keep their work and family separate and prevent interference between the two domains, thereby allowing them to experience low WFC (Kreiner, 2006; Kreiner et al., 2009; Park et al., 2011). In addition, Ashforth et al. (2000) suggested that activities that establish and manage work and family boundaries are linked to individual preferences (e.g., segmentation preferences) in the work-family interface. Based on the first and second hypotheses, we argue that employees with strong segmentation preferences regarding work and family are more likely to establish an impervious boundary to restrict the work-related use of ICT at home, which in turn enables them to experience less WFC. Thus, the following hypothesis is proposed:

**Hypothesis 3:** The work-related use of ICT at home mediates the relationship between work-family segmentation preferences and WFC.

### Group Segmentation Norms as a Group-Level Moderator

Clark (2000) argued that when employees establish boundaries between work and family, they are influenced by the boundary guard (such as managers) and other team members (such as families and coworkers) in addition to their own preferences and role identities. The effects of individual work-family segmentation preferences are affected by both formal and informal policies within their organizations (Ashforth et al., 2000). Because different working groups have different work-family segmentation norms, employees are likely to be influenced by group work-family segmentation norms, which may lead to efforts to follow the same work-family segmentation norms in their working groups. Ultimately, formal or informal segmentation and integration criteria will be established for all members. Park et al. (2011) defined group segmentation norms as the degree of consciousness of team members’ consensus of the level of work-family segmentation in working groups. Employees’ degree of consciousness about how others, such as managers and coworkers, apply the boundary between work, and family will affect employees’ own boundary establishment. According to social learning theory, people tend to imitate other members’ behaviors (Bandura, 1978; Manz and Sims, 1980).

Specifically, we argue that group segmentation norms will moderate the relationship between segmentation preferences and work-related ICT-use due to two reasons. First, employees with segmentation preferences tend to protect their valuable personal time and space by reducing the permeability and flexibility of boundaries (Derks et al., 2014). However, when employees realize that their working group expects employees to keep in touch regarding work issues after work, they may feel pressured to respond to work related calls, and emails (Barber and Santuzzi, 2015). Therefore, they will be more likely to create a relatively weak boundary between family and work in restricting the use of ICT, even if the employees are not willing to mix work with family issues, which in turn results in a high level of work to family interference. In contrast, when an employee realizes that the working group holds a strong norm of segmentation preference between work and family, s/he will establish a relatively strong boundary to restrict the use of ICT at home. Second, some research have shown that the individuals’ behavior are significantly affected by environment when the behavior is more externally motivated, even when the behavior is inconsistent with personal preferences (Cooper and Fazio, 1984). Thus, when employees with segmentation preference are pressured by group segmentation norms, they do not only adjust their sense of autonomy and control over work-related use of ICT, but also adjust the boundary of work, and family. Third, According to social identity theory and self-categorization theory (SCT), social identity is a part of the individual self-concept, including the individual’s understanding of his/her identity as a member of a social group (Turner and Reynolds, 2011). When individuals classify themselves into their working group, they will arouse group identity instead of individual identity for job issues, which shape their behavior according to the group norms. Therefore, when employees feel that the group has higher segmentation norms, they tend to adjust their segmentation preferences, and behaviors. Therefore, we propose the following hypothesis:

**Hypothesis 4A:** Group segmentation norms moderate the relationship between individual segmentation preferences and work-related use of ICT at home. Thus, when employees realize that the group has high segmentation norms, individual segmentation preferences are negatively correlated with work-related use of ICT at home more strongly than when the segmentation norms are low.

**FIGURE 1:**
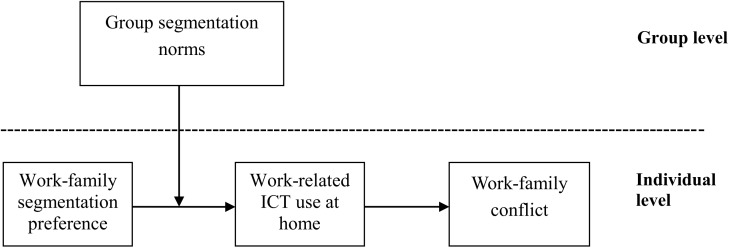
Proposed model.

Given the above hypotheses, as shown in Figure 1, we further propose an integrative mediated moderation model (Edwards and Lambert, 2007), in which we aim to test whether work-related use of ICT at home mediated the cross-level interactive effects of individual segmentation preferences, and group segmentation norms on WFC. Based on the account given in the previous sections, when employees realize that their group work-family segmentation norms are low (i.e., group members are expected to be available at home), they are less autonomous about whether use ICT at home to deal with work-related affairs or not (Derks et al., 2016). For employees with higher segmentation preference, they have to sacrifice their family time and maintain working contacts at home with their group members by using ICT, because of most other members in group handling work at home. Accordingly, they are more likely to experience higher WFC. On the contrary, when the group segmentation norms are high, employees are more autonomous on whether use ICT at home to handle work issues or not. For employees with high segmentation preference, they tend to establish boundaries for work-related ICT use in the family domain, which ensure that family affairs are not interfered by work affairs (Gadeyne et al., 2018). Accordingly, they are more likely to experience lower WFC. Therefore, we propose the following hypothesis:

**Hypothesis 4B:** Work-related use of ICT at home mediates the cross-level interactive effects of individual segmentation preferences and group segmentation norms on WFC.

## Materials and Methods

### Participants

In this study, eighty-one working groups were randomly selected from the production, technology development, marketing, and management departments of different companies. Three to ten married employees were selected from each unit to complete the questionnaire.

The enterprises in which the employees worked included a wide range of industries, such as IT, finance, electronics, and manufacturing. The self-assessment method was used in the 400 distributed questionnaires. Ultimately, 350 questionnaires were collected, and the valid response rate was as high as 87.5%. The average number of employees in each team was 4.3, 169 males, and 180 females were included, and data are missing for one respondent. The age range of the respondents was between 21 and 59, and the average age was 33.40 years.

### Measures

We used the back translation approach to ensure the reliability and validity of the scales by following previous research (e.g., Zhang et al., 2016, 2017).

*Work-family segmentation preferences* were measured using the four-item scale developed by Kreiner (2006). A sample item was the following statement: “I prefer to complete my work only during work hours.” Respondents were asked to indicate the extent to which they agreed (1, strongly disagree; 5, strongly agree) with each item. In this study, the scale reliability was 0.79.

*Work-related ICT use* was measured using the scale developed by Boswell and Olson-Buchanan (2007). Eight online communication tool used in China were assessed as representative of the ICTs commonly used by employees. Respondents were asked to report the frequency of use (1, never use; 5, frequently use) for each ICT tool, including mobile phones, QQ, fixed phones, WeChat, e-mail, and other common communication and information tools, to perform their jobs at home. The scale reliability was 0.75.

*Work-family conflict* was adapted from a five-item scale developed by Netemeyer et al. (1996). The item used a 5-point Likert scale (1, strongly disagree; 5, strongly agree). The reliability of the scale was 0.87.

*Group segmentation norm* scale was adapted from a four-item segmentation norms scale developed by Park et al. (2011). One example item is “The people I work with keep work issues at work.” A 5-point scale was adapted to segmentation norms and measured the extent to which respondents agreed with the various statements (1, strongly disagree; 5, strongly agree). In this study, the reliability of this scale was 0.77. The segmentation norm represents the attitude of a group toward work-family segmentation. For every team, the segmentation norm should be unique and stable, and all individual data must be gathered from each team to represent the entire group. Both ICC (1) and ICC (2) were tested, which are the most common indicators used to evaluate whether the convergence of individual data is reliable. To evaluate the consistency in the results, we first analyzed the differences between groups using ANOVA [*F*(80,266) = 2.18, *p* < 0.001]. The results indicated that the similarity within groups was higher than that among groups (i.e., remarkable differences were observed among groups). The calculated ICC (1) was 0.38, which is within the threshold range of 0 to 0.5 recommended by James (1982), thereby indicating that the variables had higher internal homogeneity in each group. The calculated ICC (2) was 0.71, which is greater than the critical value of 0.7 proposed by Kozlowski and Klein (2000), thereby showing that using the group-mean of the individual data as the indicator of the team variable was highly reliable. Therefore, in this study, the average score of the segmentation norms perceived by individuals in the team can be used to represent the observed team-level variables.

## Results

### Common Method Variance Test

The variables in the study were obtained through self-reporting at the same time, which may lead to common method variance. Thus, Harman’s single-factor test was conducted to identify common method variance in our study. By using exploratory factor analysis for all the variables, we examined the variance explained of the first extracted factor in the unrotated factor solution to assess common method variance. The results showed that five factors extracted in the factor analysis explained 60.62% of the total variance, and the first factor explained 22.96% of the variance, which accounted for less than 50% of the total variance explained. This result indicated that common method variance in the current study was not serious.

### Validity Test

Four confirmatory factor analyses (one-factor, two-factor, three-factor, and four-factor models) were performed to test discriminant validity among all the variables before examining the hypothesis. The hypothesized four-factor model (Model 4) had a satisfactory goodness of fit [χ^2^(183) = 308.12, *p* < 0.01, df = 183, GFI = 0.92, IFI = 0.94, CFI = 0.94, TLI = 0.93, RMSEA = 0.04], which was significantly better than that of the alternative three-factor model combining segmentation preference and segmentation norms [χ^2^(186) = 638.24, *p* < 0.01, df = 186, GFI = 0.83, IFI = 0.78, CFI = 0.78, TLI = 0.75, RMSEA = 0.08], the two-factor model combining segmentation preference, segmentation norms, and WFC [χ^2^(188) = 1056.59, *p* < 0.01, df = 188, GFI = 0.74, IFI = 0.58, CFI = 0.58, TLI = 0.53, RMSEA = 0.12] and the one-factor model [χ^2^(189) = 1239.89, *p* < 0.01, df = 189, GFI = 0.69, IFI = 0.49, CFI = 0.49, TLI = 0.43, RMSEA = 0.13]. The results showed that the measurement of all four variables had good discriminant validity.

### Descriptive Statistics and Correlation Analysis

Using the data collected from the 81 teams, descriptive statistics and correlation analyses were performed for the segmentation preferences, group segmentation norms, ICT use, and WFC. The results are summarized in Table 1. The results showed that the segmentation preferences of each employee were negatively correlated with ICT use and WFC, and ICT use was significantly positively correlated with WFC. These results provide a good basis for testing the mediation effect that will be discussed in greater detail below.

**Table 1:** Descriptive statistics and correlations.

Variable	*M*	*SD*	1	2	3	4	5	6	7	8
1. Gender	1.51	0.51	–							
2. Age	33.40	7.05	−0.14^∗^	–						
3. Work hour	45.31	10.78	−0.11^∗^	0.02	–					
4. Group size	4.74	1.47	−0.05	0.08	0.11^∗^	–				
5. Group segmentation norm^a^	3.44	0.62	0.03	0.21^∗∗^	−0.06	−0.04	0.77			
6. Work-family segmentation preference	4.05	0.70	0.16^∗∗^	−0.01	−0.17^∗∗^	−0.08	0.24^∗∗^	0.79		
7. Work-related CIT use at home	2.23	0.63	−0.11^∗^	−0.05	−0.02	0.08	−0.28^∗∗^	−0.24^∗∗^	0.75	
8. Work to family conflict	2.70	0.89	−0.27^∗∗^	0.09	0.24^∗∗^	0.16^∗^	−0.10	−0.32^∗∗^	0.30^∗∗^	0.87

*N = 350; ^∗^p < 0.05, ^∗∗^p < 0.01; ^a^The correlation value between group segmentation norm and other variables are at group-level.*

### Hypothesis Testing

For testing the proposed hypotheses above, hierarchical linear modeling (HLM) was applied to test the data. In addition, group-mean centering approach was applied to individual-level variables and grand-mean centering approach was applied to the group-level moderator before entering them into the analysis (Hofmann and Gavin, 1998). Gender, age, tenure, and working hours of the employees were included in the HLM analysis as control variables. The results are shown in Table 2. In Model 1 of Table 2, the employees’ individual work-family segmentation preferences had a significantly negative effect (γ_10_ = −0.18, *p* < 0.001) on the work-related use of ICT at home, thereby providing support for Hypothesis 1. In Model 3, ICT use at home had a significantly positive effect on WFCs (γ_20_ = 0.36, *p* < 0.001). Therefore, Hypothesis 2 was supported.

**Table 2:** Summary of hierarchical multilevel analysis (*N* = 350).

Dependent variable	Work related CIT at home	Work to family conflict				Work related CIT at home
	M1	M2	M3	M4	M5	M6
**Level 1**						
Gender	−0.12	−0.34^∗∗^	−0.34^∗∗^	−0.31^∗∗^	−0.35^∗∗^	−0.13^∗^
Age	–	0.01	0.01	0.01	0.01	–
Work hour	−0.01	0.01	0.02	0.01	0.01	−0.01
Work-family segmentation preference (γ_10_)	−0.18^∗∗^	−0.32^∗∗^		−0.26^∗∗^	−0.33^∗∗^	−0.14^∗^
Work-related CIT use at home (γ_20_)			0.36^∗∗^	0.29^∗∗^		
**Level 2**						
Group size (γ_01_)	0.04	0.06	0.06	0.05	0.07	0.05
Group segmentation norm × Individual work-family segmentation preference (γ_11_)					−0.44^∗∗^	−0.43^∗∗^
χ ^2^	202.98	161.14	132.70	138.91	177.92	231.50

*^∗^p < 0.05, ^∗∗^p < 0.01.*

Based on Model 2, the results indicated that employees’ personal segmentation preferences had a significantly negative predictive impact on their WFC (γ_10_ = −0.32, *p* < 0.01). After incorporating the personal segmentation preferences of employees and ICT use at home as predicting variables in the model, we found that the predictive effect of ICT use for work at home was remarkable (γ_20_ = 0.29, *p* < 0.01). A personal segmentation preference had a negative predictive effect on the former, whose influence coefficient (γ_10_ = −0.26, *p* < 0.01) was lower than the coefficient in Model 2 (γ_10_ = −0.31, *p* < 0.01). In addition, using the procedure proposed by Hayes and Preacher (2010), we found that the mediation effect was significant (β = −0.07, *p* < 0.01), with a 99% bootstrap confidence interval (CI) range from −0.15 to −0.03, excluding zero. Therefore, the mediation effect of individual segmentation preferences and WFC through ICT use at home was supported for Hypothesis 3.

Hypothesis 4A suggested that the group segmentation norm moderated the relationship between individual segmentation preferences and work-related ICT at home. The multilevel analysis results demonstrated that the moderation effect of the team segmentation preference norm on the relationship between the individual segmentation preference and the work-related ICT at home was significant (Model 6, γ_11_ = −0.43, *p* < 0.01). Thus, H4A was supported.

Hypothesis 4B considered the indirect effect of the interactive effect between individual work-family segmentation preferences and group segmentation norms on WFC through work-related ICT use at home. To test H4B, we first examined the moderation of group segmentation norm on the relationship between individual segmentation preference and WFC. The multilevel analysis results demonstrated the moderation was significant (Model 5, γ_11_ = −0.44, *p* < 0.01), which provided a basis for further validation of H4B. We then analyzed the interaction of group segmentation norm and individual segmentation preference on WFC whether through work-related ICT use at home. The results showed a significant indirect effect [estimate = 0.09, *p* < 0.05; 95% bootstrap (CI = −0.17, −0.03)]. Thus, H4B was also supported.

## Discussion

### Theoretical Implications

The rapid development and widespread use of ICT has allowed the interactions between work and family to develop in a novel way and has presented new opportunities and challenges for employees with respect to balancing work and family. Our results suggest that the work-family segmentation preferences of employees significantly affect the work-related use of ICT at home and reduce the degree of WFC. When employees work in a team with strong group segmentation norms, the employees’ segmentation preferences have a strong negative impact on ICT use after work. In addition to showing that employees are placed in a passive position regarding ICT use after working hours, this study suggests that an employee’s attitude toward the segmentation of work and family is an important factor that affects the home use of work-related ICT. Compared with previous studies, this study more clearly reveals the role of ICT use in managing work-family boundaries from an employee’s perspective. Previous studies have shown that the use of ICT gives employees greater control over their work and family and leads to less WFC (Valcour and Hunter, 2005), whereas other studies have suggested that ICT use integrates work and family roles, thereby blurring the boundary between these two roles, and increasing WFC (Robinson, 2006; Boswell and Olson-Buchanan, 2007). One of the main reasons for the inconsistent results is the lack of clarity regarding the control of ICT use by employees. Qualitative research conducted by Kreiner et al. (2009) found that the effects of ICT use on work-family boundary management depend on whether employees perceive that the use of ICT is under their control. Therefore, when employees are able to reach their expected level of integration or segmentation between work and family life through ICT use, boundary management between these two roles can be promoted. In addition, the inconsistent results may be caused by some moderators. Gadeyne et al. (2018) examined individual-level moderator and argue that integration preference moderates the relation between work-related ICT use and work-to-family conflict. Based on team-level analysis, we found the working group segmentation norms moderated the relation between work-related ICT use and work-to-family conflict, which further contributed ICT use research by introducing multilevel perspective.

Our research presents an analytical framework for combining moderation and mediation based on multilevel analysis. This framework clarifies how moderator variables influence the paths that constitute the direct, indirect, and total effects of mediated models (Edwards and Lambert, 2007). By using a mediated moderation model, we initially confirm that the interactive effect of weak segmentation norms and employees’ work-family segmentation preferences will impact employees’ WFC via use of ICT at home because employees in weak segmentation norms are in a passive position regarding the control of their ICT use. As a result, an individual cannot easily achieve his/her own expected level of segmentation between work and family by controlling the use of ICT, which may cause a relatively high level of WFC. In general, the results of this study confirm the significant impact of the interactive effect between employees’ characteristics and working group norms on the use of ICT after work and reveal the mechanism of ICT use in employees’ work-family boundary management, thereby providing important insights into the role of ICT use in work-family balance. In our study, we did not propose that the mediation effect varies with the change of moderator. Instead, we chose the mediated moderation model, which suggests that the path from the independent variable to the mediator (i.e., X → M) depends on the level of a moderator variable, Z, whereas the effect of the mediator on the outcome (i.e., M → Y) is constant (Hayes, 2013). We suggest that the interaction of the individual segmentation preferences and the group segmentation norms affects the mediator of the use of ICT while the effect of work-related use of ICT on the WFC is unaffected in Hypothesis 4b. This is consistent with the model structure of the mediated moderation model (e.g., Lam et al., 2007).

### Practical Implications

Our results demonstrate that an employee with a strong segmentation preference is less likely to experience WFC. Practically, the creation of more impermeable boundaries around ICT use in the family domain may be a helpful strategy for employees who experience stress associated with frequent work-family interruptions during non-work hours. In particular, employees can impose restrictions on the amount of ICT use for work during non-work hours to psychologically detach from work during leisure time. For example, they can separate their e-mail accounts, work-related social application accounts, and mobile phones for work and personal use or can utilize the selective features of the smartphone to screen incoming work-related calls during non-work time (Rothbard et al., 2005; Kreiner, 2006; Park et al., 2011; Paustian-Underdahl et al., 2016).

Second, these findings suggest that group segmentation norms moderate the relationship between work-family segmentation preferences and work-related ICT use at home. This implies that organizations should consider employees’ segmentation preferences when they design work-life balance programs.

### Limitations and Future Directions

This study has several limitations and possible future directions. First, the present study investigates the influence of work-related ICT use at home on employees’ WFC while overlooking the situation of family-related ICT use at work. In fact, it is possible for employees to use ICT to address family matters during work hours. Although active segmentation between work and family can effectively prevent WFC, it may prevent the positive spillover from work to family. Therefore, future studies should simultaneously examine employees use of ICT to manage affairs related to the family during work hours and their use of ICT to manage working affairs during non-work hours. Future studies should also explore the influence of ICT use on employees’ work and family enrichment.

Second, all the measures in our study were self-reported at the same time, which is likely to result in common method variance. Although the statistical tests demonstrated that common method variance was not serious and the data source for group segmentation norms was aggregated from multiple team members, which is different from the data source of variables at the individual level, multi-source data or an objective index should be collected in future research to improve the reliability, and validity of the construct measurements. For example, employees’ spouses can be invited to rate their work-family conflict. In addition, it is difficult for cross-sectional data to reflect the time sequence of variables, which is not conducive to examining causal relationships. Therefore, future research could collect dynamic data to examine the effect of daily work-related ICT use at home on WFC.

## Conclusion

Due to the rapid advances in communication and information technology, ICT usage continues to increase, and become more pervasive in employees’ daily lives. ICT use can help to improve employees’ productivity (Harris et al., 2015; Carlson et al., 2017), but it can also lead to increasingly permeable boundaries between work and family domains. Employees may experience more role blurring and role overload, and work-family boundary management using ICT is becoming an increasingly salient issue for employees, employers, and researchers (Park and Jex, 2011; Harris et al., 2015). There is a need to recognize the potential negative impact of work-related ICT on WFC (Harris et al., 2015; Carlson et al., 2017). This study advanced research in this area by examining individuals’ work-family segmentation preferences and organizational segmentation norms and their associations with employees’ work-related ICT during non-work time and WFC. Additionally, this study found that employees’ segmentation preferences negatively influence their psychological work to family interference through work-related ICT use, and organizational segmentation norms moderate the relationship between individuals’ segmentation preferences and ICT use for work during non-work time. Although our results extend the research in this field, we hope that these findings draw future research attention to individual-level and organizational-level boundary management work.

## Ethics Statement

The research has been performed in accordance with the recommendations of the Huaqiao University. No unethical behaviors existed in the research process. An ethics approval was not required as per applicable institutional and national guidelines and regulations. The paper and pencil survey was conducted with a research assistant present to answer any questions. In the first page of the questionnaire, we informed participants about the objectives of the study and guaranteed their confidentiality and anonymity. They were completely free to join or drop out the survey. Only those who were willing to participate were recruited. Oral and informed consent from every participant was obtained before fulfilling the survey.

## Author Contributions

JY theorized and collected the data. YZ contributed to data analysis. CS wrote the manuscript. SL contributed to the theory building. SZ contributed to literature review.

## Conflict of Interest Statement

The authors declare that the research was conducted in the absence of any commercial or financial relationships that could be construed as a potential conflict of interest.

## References

[B1] Allen T. D. Cho E. Meier L. L. (2014). Work–family boundary dynamics. *Annu. Rev. Organ. Psychol. Organ. Behav.* 1 99–121.

[B2] Ashforth B. E. Glen E. K. Fugate M. (2000). All in a days work boundaries and micro role transitions. *Acad. Manage. Rev.* 25 472–491. 10.5465/amr.2000.3363315

[B3] Bandura A. (1978). Social learning theory of aggression. *J. Commun.* 28 12–29. 10.1111/j.1460-2466.1978.tb01621.x 690254

[B4] Barber L. K. Santuzzi A. M. (2015). Please respond ASAP: workplace telepressure and employee recovery. *J. Occup. Health Psychol.* 20 172–189. 10.1037/a0038278 25365629

[B5] Boswell W. R. Olson-Buchanan J. B. (2007). The use of communication technologies after hours: the role of work attitudes and work-life conflict. *J. Manage.* 33 592–610. 10.1177/0149206307302552

[B6] Bulger C. A. Matthews R. A. Hoffman M. E. (2007). Work and personal life boundary management: boundary strength, work/personal life balance, and the segmentation-integration continuum. *J. Occup. Health Psychol.* 12 365–375. 10.1037/1076-8998.12.4.365 17953495

[B7] Carlson J. R. Carlson D. S. Zivnuska S. Harris R. B. Harris K. J. (2017). Applying the job demands resources model to understand technology as a predictor of turnover intentions. *Comput. Hum. Behav.* 77 317–325. 10.1016/j.chb.2017.09.009

[B8] Clark S. C. (2000). Work/family border theory: a new theory of work/family balance. *Hum. Relat.* 53 747–770. 10.1177/0018726700536001

[B9] Cooper J. Fazio R. H. (1984). A new look at dissonance theory. *Adv. Exp. Soc. Psychol.* 17 229–266. 10.1016/s0065-2601(08)60121-5 12709184

[B10] Derks D. Bakker A. B. Peters P. Wingerden P. V. (2016). Work-related smartphone use, work-family conflict and family role performance: the role of segmentation preference. *Hum. Relat.* 69 1045–1068. 10.1177/0018726715601890

[B11] Derks D. van Mierlo H. Schmitz E. B. (2014). A diary study on work-related smartphone use, psychological detachment and exhaustion: examining the role of the perceived segmentation norm. *J. Occup. Health Psychol.* 19 74–84. 10.1037/a0035076 24447222

[B12] Edwards J. R. Lambert L. S. (2007). Methods for integrating moderation and mediation: a general analytical framework using moderated path analysis. *Psychol. Methods* 12 1–22. 10.1037/1082-989x.12.1.1 17402809

[B13] Fenner G. H. Renn R. W. (2004). Technology-assisted supplemental work: construct definition and a research framework. *Hum. Resourc. Manage.* 43 179–200. 10.1002/hrm.20014

[B14] Ferguson M. Carlson D. Boswell W. Whitten D. Butts M. M. Kacmar K. M. (2016). Tethered to work: a family systems approach linking mobile device use to turnover intentions. *J. Appl. Psychol.* 101 520–534. 10.1037/apl0000075 26653530

[B15] Gadeyne N. Verbruggen M. Delanoeije J. De Cooman R. (2018). All wired, all tired? Work-related ICT-use outside work hours and work-to-home conflict: the role of integration preference, integration norms and work demands. *J. Vocat. Behav.* 107 86–99. 10.1016/j.jvb.2018.03.008

[B16] Gephart R. P. Jr. (2002). Introduction to the brave new workplace: organizational behavior in the electronic age. *J. Organ. Behav.* 23 327–344. 10.1002/job.143

[B17] Grant L. Kinman G. (2014). *Developing Resilience for Social Work Practice.* Basingstoke: Palgrave Macmillan.

[B18] Greenhaus J. H. Beutell N. J. (1985). Sources of conflict between work and family roles. *Acad. Manage. Rev.* 10 76–88. 10.5465/amr.1985.4277352

[B19] Halbesleben J. R. Harvey J. Bolino M. C. (2009). Too engaged? A conservation of resources view of the relationship between work engagement and work interference with family. *J. Appl. Psychol.* 94 1452–1465. 10.1037/a0017595 19916655

[B20] Halbesleben J. R. Neveu J. P. Paustian-Underdahl S. C. Westman M. (2014). Getting to the “COR” understanding the role of resources in conservation of resources theory. *J. Manage.* 40 1334–1364. 10.1177/0149206314527130

[B21] Harris K. J. Harris R. B. Carlson J. R. Carlson D. S. (2015). Resource loss from technology overload and its impact on work-family conflict: can leaders help? *Comput. Hum. Behav.* 50 411–417. 10.1016/j.chb.2015.04.023

[B22] Hayes A. F. (2013). Introduction to mediation, moderation, and conditional process analysis: a regression-based approach. *J. Educ. Meas.* 51 335–337. 28385036

[B23] Hayes A. F. Preacher K. J. (2010). Quantifying and testing indirect effects in simple mediation models when the constituent paths are nonlinear. *Multivariate Behav. Res.* 45 627–660. 10.1080/00273171.2010.498290 26735713

[B24] Hendon M. Powell L. Wimmer H. (2017). Emotional intelligence and communication levels in information technology professionals. *Comput. Hum. Behav.* 71 165–171. 10.1177/0969733015594665 26208722

[B25] Higgins C. Thomas J. Duxbury L. Towers I. Carr A. N. (2006). Time thieves and space invaders: technology, work and the organization. *J. Organ. Chang. Manage.* 19 593–618. 10.1108/09534810610686076

[B26] Hislop D. Axtell C. Collins A. Daniels K. Glover J. Niven K. (2015). Variability in the use of mobile ICTs by homeworkers and its consequences for boundary management and social isolation. *Inform. Organ.* 25 222–232. 10.1016/j.infoandorg.2015.10.001

[B27] Hobfoll S. E. (1989). Conservation of resources: a new attempt at conceptualizing stress. *Am. Psychol.* 44 513–524. 10.1037//0003-066x.44.3.513 2648906

[B28] Hobfoll S. E. Shirom A. (2001). “Conservation of resources theory: applications to stress and management in the workplace,” in *Handbook of Organizational Behavior*, ed. Golembiewski R. T. (New York, NY: Marcel Dekker), 57–80

[B29] Hofmann D. A. Gavin M. B. (1998). Centering decisions in hierarchical linear models: implications for research in organizations. *J. Manage.* 24 623–641. 10.1016/s0149-2063(99)80077-4

[B30] Ilies R. Wilson K. S. Wagner D. T. (2009). The spillover of daily job satisfaction onto employees’ family lives: the facilitating role of work-family integration. *Acad. Manage. J.* 52 87–102. 10.5465/amj.2009.36461938

[B31] James L. R. (1982). Aggregation bias in estimates of perceptual agreement. *J. Appl. Psychol.* 67 219–229. 10.1037/0021-9010.67.2.219

[B32] Kossek E. E. Lautsch B. A. (2012). Work–family boundary management styles in organizations: a cross-level model. *Organ. Psychol. Rev.* 2 152–171. 10.1177/2041386611436264

[B33] Kossek E. E. Lautsch B. A. Eaton S. C. (2006). Telecommuting, control, and boundary management: correlates of policy use and practice, job control, and work–family effectiveness. *J. Vocat. Behav.* 68 347–367. 10.1016/j.jvb.2005.07.002

[B34] Kozlowski S. W. Klein K. J. (2000). “A multilevel approach to theory and research in organizations: contextual, temporal, and emergent processes,” in *Multilevel Theory, Research, and Methods in Organizations*, eds Kozlowski S. W. Klein K. J. (San Francisco, CA: Jossey-Bass), 3–90.

[B35] Kreiner G. E. (2006). Consequences of work-home segmentation or integration: a person-environment fit perspective. *J. Organ. Behav.* 27 485–507. 10.1002/job.386 17401684

[B36] Kreiner G. E. Hollensbe E. C. Sheep M. L. (2009). Balancing borders and bridges: negotiating the work-home interface via boundary work tactics. *Acad. Manage. J.* 52 704–730. 10.5465/amj.2009.43669916

[B37] Lam W. Huang X. Snape E. (2007). Feedback-seeking behavior and leader-member exchange: do supervisor-attributed motives matter? *Acad. Manage. J.* 50 348–363. 10.5465/amj.2007.24634440

[B38] Liao Y. Yang Z. Wang M. Kwan H. K. (2016). Work–family effects of LMX: the moderating role of work–home segmentation preferences. *Leadersh. Quart.* 27 671–683. 10.1016/j.leaqua.2016.03.003

[B39] Liu J. Kwan H. K. Lee C. Hui C. (2013). Work-to-family spillover effects of workplace ostracism: the role of work-home segmentation preferences. *Hum. Resourc. Manage.* 52 75–93. 10.1002/hrm.21513

[B40] Major D. A. Germano L. M. (2006). “The changing nature of work and its impact on the work-home interface,” in *Work-Life Balance: A Psychological Perspective*, eds F. Jones Burke R. J. Westman M. (New York, NY: Psychology Press), 13–38.

[B41] Manz C. C. Sims H. P. Jr. (1980). Self-management as a substitute for leadership: a social learning theory perspective. *Acad. Manage. Rev.* 5 361–367. 10.5465/amr.1980.4288845

[B42] Matthews R. A. Barnes-Farrell J. L. (2010). Development and initial evaluation of an enhanced measure of boundary flexibility for the work and family domains. *J. Occup. Health Psychol.* 15 330–346. 10.1037/a0019302 20604638

[B43] Middleton C. A. (2007). Illusions of balance and control in an always-on environment: a case study of Black Berry users. *Continuum* 21 165–178. 10.1080/10304310701268695

[B44] Milliken F. J. Dunnjensen L. M. (2005). “The changing time demands of managerial and professional work: implications for managing the work-life boundary,” in *LEA’s Organization and Management Series. Work and Life Integration: Organizational, Cultural, and Individual Perspectives*, eds Kossek E. E. Lambert S. J. (Mahwah, NJ: Lawrence Erlbaum Associates Publishers), 43–59.

[B45] Netemeyer R. G. Boles J. S. McMurrian R. (1996). Development and validation of work–family conflict and family–work conflict scales. *J. Appl. Psychol.* 81 400–410. 10.1037//0021-9010.81.4.400

[B46] Ninaus K. Diehl S. Terlutter R. Chan K. Huang A. (2015). Benefits and stressors – perceived effects of ICT use on employee health and work stress: an exploratory study from Austria and Hong Kong. *Int. J. Qual. Stud. Health Well Being* 10:28838. 10.3402/qhw.v10.28838 26462972 PMC4604212

[B47] Olson-Buchanan J. B. Boswell W. R. (2006). Blurring boundaries: correlates of integration and segmentation between work and nonwork. *J. Vocat. Behav.* 68 432–445. 10.1016/j.jvb.2005.10.006

[B48] Park Y. Fritz C. Jex S. M. (2011). Relationships between work-home segmentation and psychological detachment from work: the role of communication technology use at home. *J. Occup. Health Psychol.* 16 457–467. 10.1037/a0023594 21728434

[B49] Park Y. Jex S. M. (2011). Work-home boundary management using communication and information technology. *Int. J. Stress Manage.* 18 133–152. 10.1037/a0022759

[B50] Paustian-Underdahl S. C. Halbesleben J. R. Carlson D. S. Kacmar K. M. (2016). The work–family interface and promotability: boundary integration as a double-edged sword. *J. Manage.* 42 960–981. 10.1177/0149206313506464

[B51] Powell G. N. Greenhaus J. H. (2010). Sex, gender, and the work-to-family interface: exploring negative and positive interdependencies. *Acad. Manage. J.* 53 513–534. 10.5465/amj.2010.51468647

[B52] Robinson J. (2006). An e-tool bill of rights. *Fast Company* 111:54.

[B53] Rothbard N. P. Phillips K. W. Dumas T. L. (2005). Managing multiple roles: work-family policies and individuals’ desires for segmentation. *Organ. Sci.* 16 243–258. 10.1287/orsc.1050.0124

[B54] Towers I. Duxbury L. Higgins C. Thomas J. (2006). Time thieves and space invaders: technology, work and the organization. *J. Organ. Chang. Manage.* 19 593–618. 10.1108/09534810610686076

[B55] Turner J. C. Reynolds K. J. (2011). Self-categorization theory. *Handb. Theor. Soc. Psychol.* 2 399–417. 10.4135/9781446201022.n46

[B56] Valcour P. M. Hunter L. W. (2005). “Technology, organizations, and work-life integration,” in *LEA’s Organization and Management Series. Work and Life Integration: Organizational, Cultural, and Individual Perspectives*, eds Kossek E. E. Lambert S. J. (Mahwah, NJ: Lawrence Erlbaum Associates Publishers), 61–84.

[B57] Zhang L. Zhang Y. Jiang H. Yang M. M. Huang Y.-Y. Li S.-J. (2017). Customer identification in the healthcare industry. *Int. J. Mark. Res.* 59 1–20. 10.2501/IJMR-2017-054

[B58] Zhang Y. Zhang L. Lei H. Yue Y. Zhu J. (2016). Lagged effect of daily surface acting on subsequent day’s fatigue. *Serv. Ind. J.* 36 809–826. 10.1080/02642069.2016.1272593 30975987

[B59] Zimmerman R. D. Swider B. W. Woo S. E. Allen D. G. (2016). Who withdraws? Psychological individual differences and employee withdrawal behaviors. *J. Appl. Psychol.* 101 498–519. 10.1037/apl0000068 26595754

